# Stereotactic ablative radiotherapy versus conventional fractionated radiotherapy for clinical early‐stage non‐small‐cell lung cancer: a population‐based study

**DOI:** 10.1111/1759-7714.15404

**Published:** 2024-07-16

**Authors:** Hung‐Jen Chen, Wen‐Chien Cheng, Chih‐Yen Tu, Te‐Chun Hsia, Yu‐Sen Lin, Hsin‐Yuan Fang, Chia‐Chin Li, Chun‐Ru Chien

**Affiliations:** ^1^ Division of Pulmonary and Critical Care Medicine, Department of Internal Medicine China Medical University Hospital Taichung Taiwan; ^2^ School of Medicine, College of Medicine China Medical University Taichung Taiwan; ^3^ Ph.D. Program for Health Science and Industry, College of Health Care China Medical University Taichung Taiwan; ^4^ Department of Chest Surgery China Medical University Hospital Taichung Taiwan; ^5^ Department of Radiation Oncology China Medical University Hospital Taichung Taiwan

**Keywords:** conventional fractionated radiotherapy, non‐small‐cell lung cancer, stereotactic ablative radiotherapy

## Abstract

**Introduction:**

The use of stereotactic ablative radiotherapy (SABR) over conventional fractionated radiotherapy (CFRT) for early‐stage non‐small‐cell lung cancer (NSCLC) has been advocated, but is also debated in the literature.

**Methods:**

In this retrospective cohort study, we adopted a target trial emulation framework to identify eligible patients diagnosed between 2011 and 2021 using the Taiwan Cancer Registry. In the primary analysis, the overall survival (OS) was the primary endpoint, whereas incidences of lung cancer mortality and radiation pulmonary toxicity were the secondary endpoints. Extensive supplementary analyses were also conducted.

**Results:**

We included 351 patients in the primary analysis and found that the OS was not significantly different between the SABR (*n* = 290) and CFRT (*n* = 61) groups. The propensity score weighting adjusted hazard ratio of death was 0.75 (95% confidence interval 0.53–1.07, *p* = 0.118). The secondary endpoints and supplementary analyses showed no significant differences.

**Conclusions:**

The OS of patients with early‐stage NSCLC treated with SABR was not significantly different from that of patients treated with CFRT alone. The results of the relevant ongoing clinical trials are eagerly awaited.

## Graphical abstract

The overall survival of patients with early‐stage non‐small‐cell lung cancer treated with stereotactic ablative radiotherapy was not significantly different from that of patients treated with conventional fractionated radiotherapy alone.

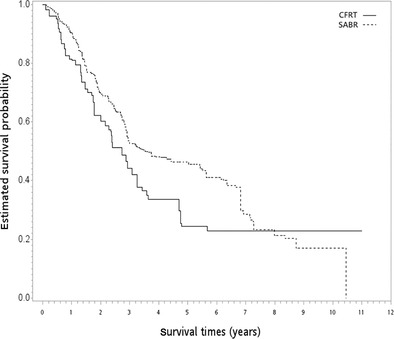

## INTRODUCTION

Lung cancer is a leading cause of cancer mortality around the world, including in Taiwan.^1^, ^2^ Most cases include non‐small‐cell lung cancer (NSCLC),^1^, ^2^ where surgery is the treatment of choice for early‐stage disease.^1^, ^3^ However, radiotherapy is indicated for cases not suitable for surgery, wherein stereotactic ablative radiotherapy (SABR) or stereotactic body radiotherapy (SBRT) is preferred over conventional fractionated radiotherapy (CFRT).^1^, ^4^ The superiority of SABR over CFRT might be related to the higher biologically effective dose (BED) usually administered for SABR.^4^, ^5^, ^6^

However, a systematic review published in 2023 challenged the superiority of SABR.^7^ Based on two published randomized controlled trials (RCTs),^8^, ^9^ it stated that “Despite widespread adoption and extensive single‐arm prospective and retrospective studies suggesting its benefit, this systematic review and meta‐analysis of randomized trials fail to confirm improvements in local control, overall survival (OS), and toxicity profile of SABR over CFRT in early NSCLC.” These findings are consistent with the primary analyses in our previous retrospective study published in 2017.^10^

Considering the aforementioned debate comparing the efficacy of SABR to that of CFRT in early‐stage NSCLC, and the scarcity of retrospective studies from Asia,^11^ we conducted this updated retrospective cohort study utilizing the Taiwan Cancer Registry (TCR) and the target trial emulation framework.^12^, ^13^ This study includes more recent patients and an extended follow‐up period.

## MATERIALS AND METHODS

### Data source

The Health and Welfare Data Science Center (HWDC) database, with personal identifiers removed, includes complete information regarding the TCR, death registration, and reimbursement data for the entire Taiwanese population provided by the Bureau of National Health Insurance. The TCR is one of the highest quality cancer registries worldwide.^14^ This study was approved by the Central Regional Research Ethics Committee of China Medical University, Taichung, Taiwan (CRREC‐108‐080 [CR‐4]).

### Study design, population, and intervention

In this retrospective cohort study, we adopted the target trial emulation framework.^12^, ^13^, ^15^ Our target trial design was modified from available RCTs^7^, ^8^, ^9^ and the National Comprehensive Cancer Network guidelines^4^ (see Table 1 for details of the study design).

**TABLE 1:** Target trial protocol^15^.

Protocol elements	Target trial^a^	Emulation with data from TCR
Eligibility criteria	Age ≥18 years; NSCLC stage (AJCC 7th T1‐2aN0M0) or (AJCC 8th T1‐2N0M0); ECOG PS 0–2; unresected; without prior cancer, radiotherapy, or systemic therapy	Age ≥18 years; NSCLC stage (AJCC 7th T1‐2aN0M0) or (AJCC 8th T1‐2N0M0); ECOG PS 0–2; received curative therapy without surgery; no previous cancer history in TCR
Treatment strategies	SABR: BED(10) ≥87.5 Gy_10_ with total fractions ≤10 and dose per fraction ≥6 Gy; CFRT: BED(10) within 62.5–84 Gy_10_ with dose per fraction within 2–2.5 Gy	Same as target trial
Treatment assignment	Randomization, no blinding	Eligible individuals are assigned at baseline to the treatment strategy consistent with their data To emulate randomization, adjustments are made for baseline confounders (see subsection “Covariates” in the Section Materials and Methods for details)
Outcomes	Primary outcome: OS; Secondary outcome: ILCM	Same as target trial
Causal estimand	ITT effect in primary analyses; PP effect in supplementary analyses	Same as target trial (using start of radiotherapy as surrogate of ITT)
Start and end of follow‐up	Starts at randomization and ends at occurrence of end point or follow‐up	Starts at radiotherapy initiation and ends at occurrence of end point or administrative censoring
Statistical analysis	Log‐rank test and Cox proportional hazards regression	Hazard ratios are estimated using Cox proportional hazards model in the weighted sample while adjusting for baseline confounders using the overlap weight

Abbreviations: AJCC, American Joint Committee on Cancer; BED, biologically effective dose^5^; CFRT, conventional fractionated radiotherapy; ECOG PS, Eastern Cooperative Oncology Group performance status; ILCM, incidence of lung cancer mortality; ITT, intention‐to‐treat; NSCLC, non‐small‐cell lung cancer; OS, overall survival; PP, per protocol; SABR, stereotactic ablative radiotherapy; TCR, Taiwan Cancer Registry.

^a^ Modified from SPACE^8^ and CHISEL,^9^ and the National Comprehensive Cancer Network NSCLC 2024v1 treatment guidelines.^4^

We identified patients with NSCLC who met the following inclusion criteria: tumor staging according to the American Joint Committee on Cancer (AJCC) (7th edition, T1–2aN0M0 or 8th edition, T1–2N0M0, with tumor size ≤5 cm in both editions), age ≥18 years,^16^, ^17^ diagnosed between 2011 and 2021, and received curative radiotherapy without surgery. Patients were further classified as the SABR group (fractional [Fx] dose ≥6 Gy/Fx, total fractions ≤10 Fx, and total BED (10) ≥87.5) or the CFRT group (fractional dose within 2 − ≤2.5 Gy/Fx and total BED (10) within 62.5–84). The choice of these cutoff doses/fractionations was based on guidelines and pivotal trials.^4^, ^9^

**FIGURE 1:**
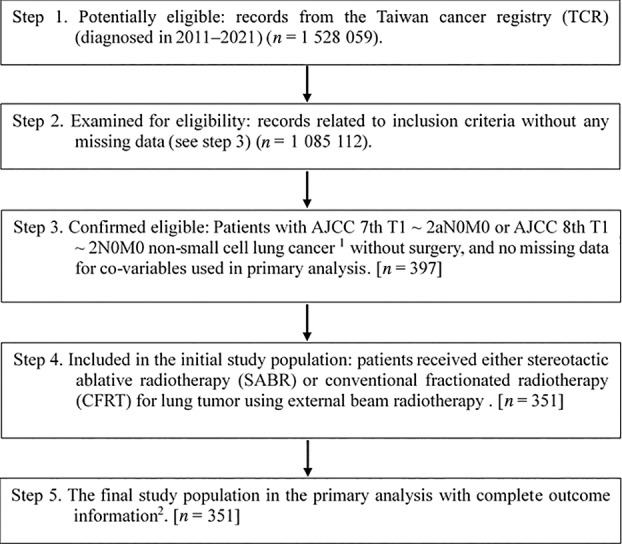
STROBE study flowchart and number of individuals at each stage of the study. ^1^To ensure data consistency, only treated patients (class 1–2) and those with a single record were included. ^2^Without missing information regarding survival status in the TCR and death registry.

The primary outcome of interest was OS. Incidences of lung cancer mortality (ILCM) and radiation pulmonary toxicity were considered secondary outcomes. These outcomes were evaluated through linkages with the TCR, death registry, or reimbursement records. The first radiotherapy session date was defined as the index date, and the OS/ILCM was calculated from the index date to the date of death or until December 31, 2022 (the censoring date of the death registry). The study flowchart, as suggested by the STROBE guidelines,^18^ is shown in Figure 1.

### Covariates

We collected related covariates based on recent relevant studies and our clinical research experiences^8^, ^9^, ^10^ to adjust for potential nonrandomized treatment selection. Patient demographics (age, sex, residency region, social‐economic status [SES], and diagnosis period), patient characteristics (comorbidity, performance status, and smoking history), disease characteristics (histology, clinical T‐stage, use of positron emission tomography [PET] at diagnosis, and tumor size), and treatment characteristics (use of systemic therapy) were defined as follows. Age (years) and tumor size (mm) were treated as continuous variables. Sex was classified as either male or female. The region of patient residence in Taiwan was classified as north and non‐north. SES was classified as an income equivalent to minimum wage or lower, or higher than minimum wage. Comorbidity was determined using the Charlson comorbidity index score and classified as ≥1 or <1 as per modifications from the literature.^19^ Histology was classified as adenocarcinoma or non‐adenocarcinoma. The study period was set as 2011–2017 or 2018–2021 owing to changes in the AJCC clinical staging criteria since 2018. The clinical T stage was classified as T1 or T2. History of PET, systemic therapy, and smoking was classified as yes or no. Eastern Cooperative Oncology Group (ECOG) performance status was scored as 0–1 or 2.

### Statistical and supplementary analyses

In the primary analysis, the propensity score (PS) weighting (PSW) approach with an overlap weight was used to balance the observed covariates and emulate randomization.^20^, ^21^, ^22^, ^23^, ^24^ We used a logistic regression model with the above‐mentioned covariates to estimate PS.^24^ After PSW, the standardized difference was used to assess the covariate balance between groups.^25^, ^26^ We then compared the hazard ratio (HR) of death between the groups during the entire follow‐up period. We analyzed all patients who initiated SABR (fractional dose ≥6 Gy) or CFRT (fractional dose within 2–2.5 Gy) regardless of the received biological dose to mimic the intention‐to‐treat (ITT) analyses. Point estimation was calculated using the Cox proportional hazards model for the weighted sample, and the 95% confidence interval (95% CI) was estimated using the bootstrap method.^27^, ^28^, ^29^, ^30^ Regarding potential unmeasured confounders, we used the E‐value to assess the robustness of our findings.^31^, ^32^ The incidence of ILCM between groups was evaluated using a competing risk approach in the weighted sample.^33^ We used the International Classification of Diseases, Ninth Revision, Clinical Modification (ICD‐9‐CM) and ICD‐10‐CM coding (5080/5081 and J700/J701, respectively^34^) to estimate the occurrence of radiation pulmonary toxicity within 6 months of radiotherapy.^22^

In the supplementary analyses (SA), we conducted five additional analyses to evaluate the robustness of our findings. SA‐1 was a per‐protocol (PP) analyses including only those patients who received the intended dose (BED (10) ≥87.5 and fraction number ≤10 for SABR or BED (10) within 62.5–84 for CFRT) with the same analytic approach as in the primary analyses. SA‐2 was an alternative analytic approach, including propensity score matching (PSM)^20^, ^24^ within the SA‐1 study population to construct a 1:1 PS‐matched cohort (without replacement). We compared the HR using a robust variance estimator^27^ and adopted the sub‐distribution HR to assess ILCM employing the clustered Fine–Gray model.^35^ We compared the rate of radiation pulmonary toxicity using McNemar's test.^36^ In SA‐3, we limited the radiotherapy dose/fractionation for the two groups (SABR [BED (10) ≥100 Gy] vs. CFRT [BED (10) within 72–84 Gy]) in the SA‐1 study population to explore the difference between them when a more radical radiotherapy dose^6^, ^8^, ^37^ was used. In SA‐4, we limited the analysis to patients in the SA‐1 study population potentially compatible with the national health insurance criteria for SABR (medically inoperable), according to TCR records. In SA‐5, we considered urinary tract infection (UTI), modified from the literature^38^ and not expected to be associated with thoracic radiotherapy, as the negative control outcome.^39^ UTI occurrence post thoracic radiotherapy was then compared within the SA‐1 study population using methods suggested in the literature.^22^

Statistical analyses were performed using SAS software (version 9.4; SAS Institute) and R (version 4.3.0; R Development Core Team, R Foundation for Statistical Computing).

## RESULTS

### Study population

As shown in Figure 1, 351 eligible patients with clinical early‐stage NSCLC who received either SABR (290 patients) or CFRT (61 patients) between 2011 and 2021 were included in the primary analysis. Patient characteristics are described in Table 2. All covariates were well balanced (standardized differences ≈ 0) after PSW with overlap weights, although some (sex, residency region, SES, tumor size, period, T stage, use of PET, use of systemic therapy, performance status) were imbalanced before PSW.^25^

**TABLE 2:** Patient characteristics for the whole study population in the primary analysis.

	SABR (*n* = 290)		CFRT (*n* = 61)		Standardized difference^a^	
	Number or mean (SD)^a^	(%)^a^	Number or mean (SD)^a^	(%)^a^	Before PSW	After PSW
Age (years)	77.76 (9.36)		76.97 (8.80)		0.087	≈0
Sex						
Female	121	(42)	16	(26)	0.332	≈0
Male	169	(58)	45	(74)		
Residency region						
Non‐north	142	(49)	38	(62)	0.271	≈0
North	148	(51)	23	(38)		
Social‐economic status						
Minimum wage or lower	125	(43)	18	(30)	0.286	≈0
Higher	165	(57)	43	(70)		
Comorbidity						
<1	46	(16)	12	(20)	0.100	≈0
≥1	244	(84)	49	(80)		
Smoking						
No	139	(48)	23	(38)	0.208	≈0
Yes	151	(52)	38	(62)		
Histology						
Non‐adenocarcinoma	94	(32)	26	(43)	0.212	≈0
Adenocarcinoma	196	(68)	35	(57)		
T stage						
T1	159	(55)	18	(30)	0.530	≈0
T2	131	(45)	43	(70)		
Period						
2011–2017	127	(44)	42	(69)	0.522	≈0
2018–2021	163	(56)	19	(31)		
Use of PET						
No	84	(29)	35	(57)	0.599	≈0
Yes	206	(71)	26	(43)		
Use of systemic therapy						
No	271	(93)	44	(72)	0.589	≈0
Yes	19	(7)	17	(28)		
Performance status						
0–1	215	(74)	52	(85)	0.279	≈0
2	75	(26)	9	(15)		
Tumor size (mm)	2.87 (0.97)		3.14 (0.93)		0.282	≈0

Abbreviations: CFRT, conventional fractionated radiotherapy; PET, positron emission tomography; PSW, propensity score weighting; SABR, stereotactic ablative radiotherapy; SD, standard deviation.

^a^ Rounded.

### Primary analysis

**FIGURE 2:**
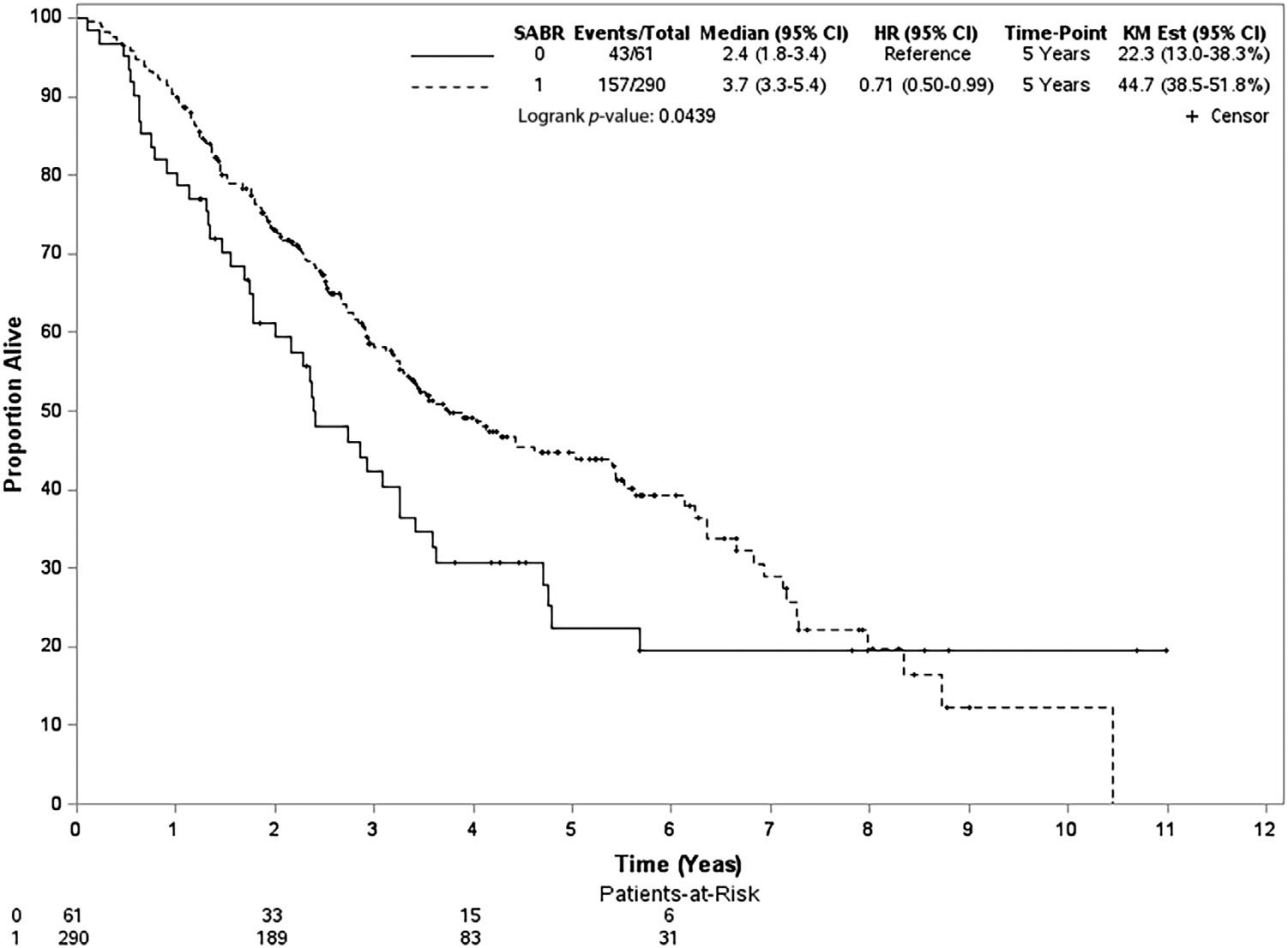
Unadjusted Kaplan–Meier survival curve for SABR versus CFRT in the whole study population. CFRT, conventional fractionated radiotherapy; SABR, stereotactic ablative radiotherapy.

**FIGURE 3:**
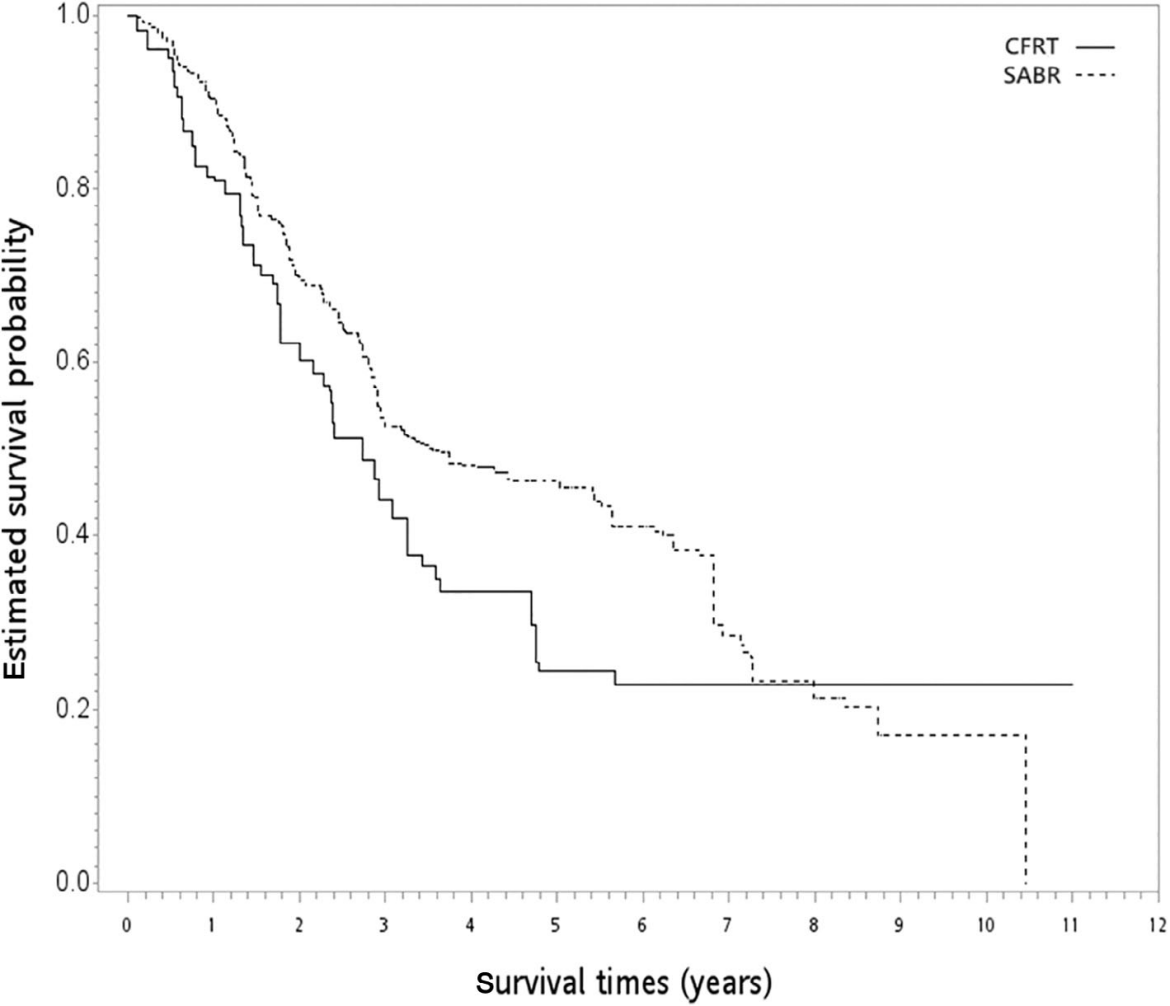
PSW‐adjusted Kaplan–Meier survival curve for SABR versus CFRT in the whole study population. CFRT, conventional fractionated radiotherapy; PSW, propensity score weighting; SABR, stereotactic ablative radiotherapy.

After a median follow‐up of 31 months (range = 1–132 months), 200 deaths were observed (157 and 43 patients in the SABR and CFRT groups, respectively). In the unadjusted analysis, the 5‐year OS rates were 22% (CFRT group) and 45% (SABR group) (log‐rank test, *p* = 0.044) (Figure 2). The PSW‐adjusted OS curve is shown in Figure 3. The 5‐year PSW‐adjusted OS rates were 24% (CFRT group) and 46% (SABR group). When comparing the SABR and CFRT groups, the PSW‐adjusted HR of death was 0.75 (95% CI 0.53–1.07, *p* = 0.118). This observed value could be explained by an unmeasured confounder associated with the selected treatment (CFRT or SABR) and survival with a risk ratio of 1.74 (E‐value)‐fold each. However, weaker confounding factors could not explain this association. Furthermore, the result for ILCM (HR = 0.68, *p* = 0.226) and the rate of radiation pulmonary toxicity after radiotherapy (<5%, *p* = 0.664) were not significantly different between the groups, but the details were not reported owing to HWDC data policy.

### Supplementary analyses

**FIGURE 4:**
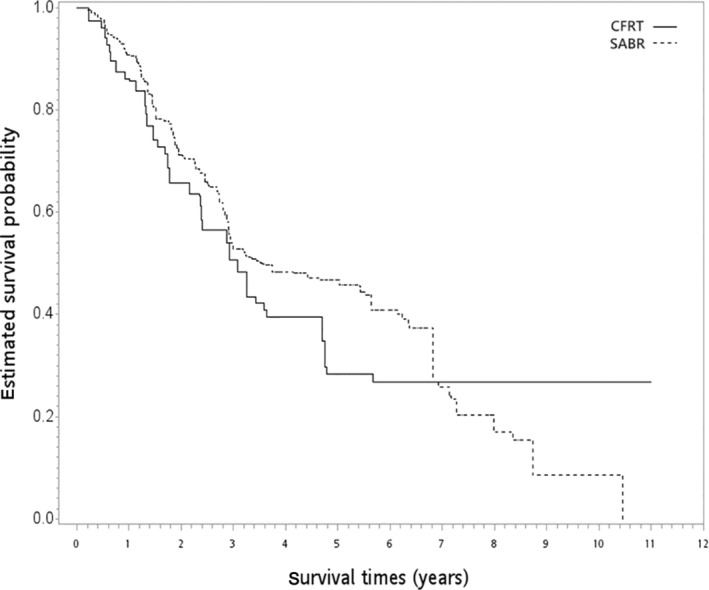
PSW‐adjusted Kaplan–Meier survival curve for SABR versus CFRT in the first supplementary analysis. CFRT, conventional fractionated radiotherapy; PSW, propensity score weighting; SABR, stereotactic ablative radiotherapy.

In the SA‐1 group, 270 and 53 patients in the SABR and CFRT groups received per‐protocol radiotherapy doses, respectively. Patient characteristics are described in Table 3. All covariates achieved a good balance after PSW with overlapping weights. When the SABR group was compared with the CFRT group, the PSW‐adjusted HR of death was 0.88 (95% CI 0.60–1.30, *p* = 0.520). The PSW‐adjusted OS curves are shown in Figure 4. The result for ILCM remained insignificant between the groups (HR = 0.69, *p* = 0.287). The rate of radiation pulmonary toxicity was similarly low (<5%) in both groups, but the details were not reported due to the HWDC data policy.

**TABLE 3:** Patient characteristics for the study population in the first supplementary analysis.

	SABR (*n* = 270)		CFRT (*n* = 53)		Standardized difference^a^	
	Number or mean (SD)^a^	(%)^a^	Number or mean (SD)^a^	(%)^a^	Before PSW	After PSW
Age (years)	77.90 (9.20)		77.30 (8.00)		0.069	≈0
Sex						
Female	111	(41)	13	(25)	0.359	≈0
Male	159	(59)	40	(75)		
Residency region						
Non‐north	134	(50)	34	(64)	0.296	≈0
North	136	(50)	19	(36)		
Social‐economic status						
Minimum wage or lower	117	(43)	15	(28)	0.317	≈0
Higher	153	(57)	38	(72)		
Comorbidity						
<1	42	(16)	10	(19)	0.088	≈0
≥1	228	(84)	43	(81)		
Smoking						
No	126	(47)	21	(40)	0.143	≈0
Yes	144	(53)	32	(60)		
Histology						
Non‐adenocarcinoma	89	(33)	22	(42)	0.177	≈0
Adenocarcinoma	181	(67)	31	(58)		
T stage						
T1	146	(54)	16	(30)	0.499	≈0
T2	124	(46)	37	(70)		
Period						
2011–2017	114	(42)	36	(68)	0.535	≈0
2018–2021	156	(58)	17	(32)		
Use of PET						
No	77	(29)	30	(57)	0.592	≈0
Yes	193	(71)	23	(43)		
Use of systemic therapy						
No	254	(94)	38	(72)	0.622	≈0
Yes	16	(6)	15	(28)		
Performance status						
0–1	200	(74)	47	(89)	0.382	≈0
2	70	(26)	6	(11)		
Tumor size (mm)	2.88 (0.97)		3.14 (0.97)		0.268	≈0

Abbreviations: CFRT, conventional fractionated radiotherapy; PET, positron emission tomography; PSW, propensity score weighting; SABR, stereotactic ablative radiotherapy; SD, standard deviation.

^a^ Rounded.

**FIGURE 5:**
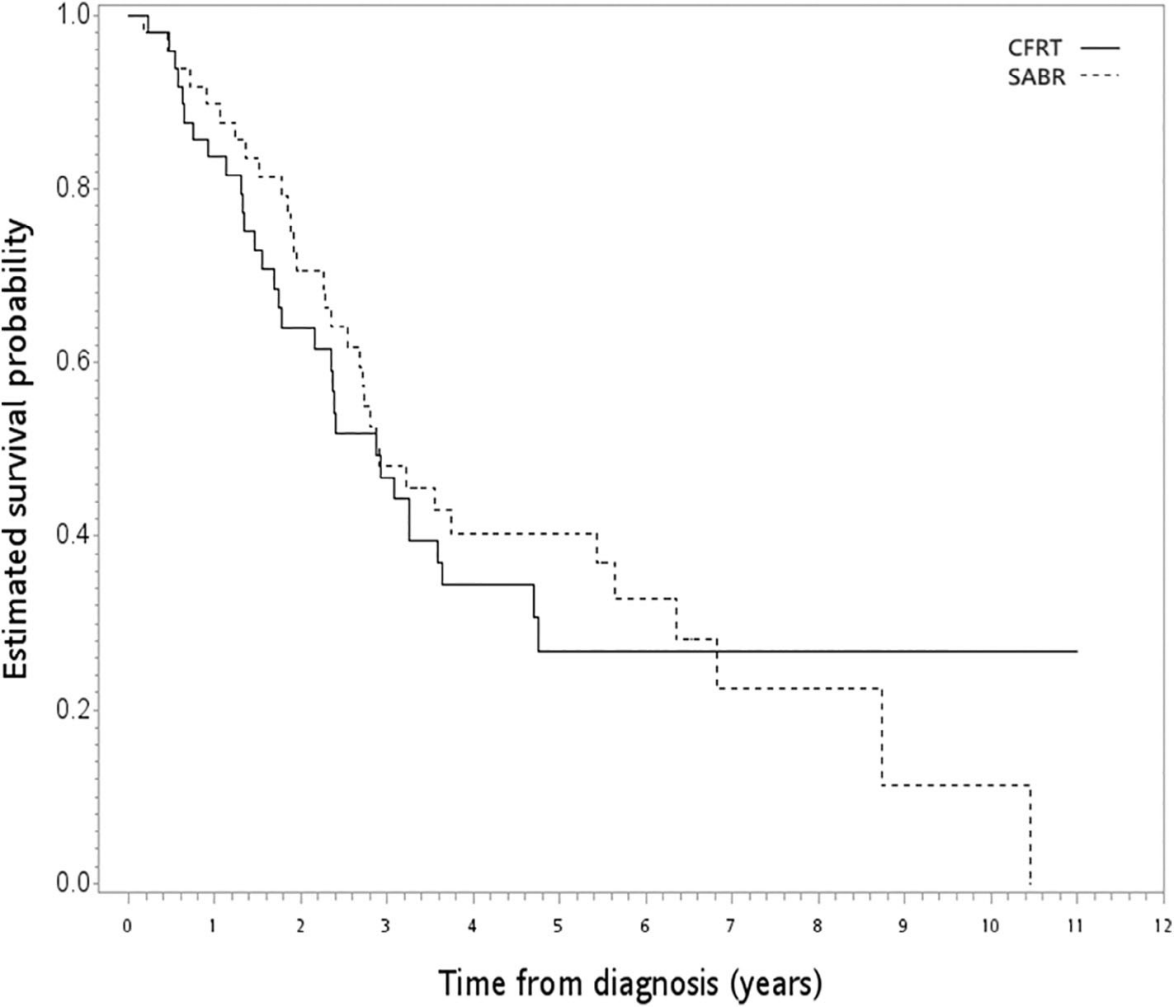
Kaplan–Meier survival curve for SABR versus CFRT in the second supplementary analysis. CFRT, conventional fractionated radiotherapy; SABR, stereotactic ablative radiotherapy.

In SA‐2, the constructed PS‐matched subgroup (*n* = 98) with a good balance of covariates after PSM is presented in Table 4. The Kaplan–Meier OS curve is shown in Figure 5. The 5‐year OS rates were 27% (CFRT group) and 40% (SABR group). Compared to CFRT, the OS (HR = 0.94, 95% CI 0.56–1.59, *p* = 0.829) and ILCM (HR = 0.71, *p* = 0.230) of patients receiving SABR had no significant difference between the groups. The rate of radiation pulmonary toxicity was similarly low (<5%) in both groups. The details were not reported due to the HWDC data policy.

**TABLE 4:** Patient characteristics for the study population in the second supplementary analysis.

	SABR (*n* = 49)		CFRT (*n* = 49)		
	Number or mean (SD)^a^	(%)^a^	Number or mean (SD)^a^	(%)^a^	Standardized difference^a^
Age (years)	77.39 (10.54)		77.02 (8.19)		0.039
Sex					
Female	14	(29)	13	(27)	0.046
Male	35	(71)	36	(73)	
Residency region					
Non‐north	26	(53)	31	(63)	0.208
North	23	(47)	18	(37)	
Social‐economic status					
Minimum wage or lower	15	(31)	14	(29)	0.045
Higher	34	(69)	35	(71)	
Comorbidity					
<1	9	(18)	10	(20)	0.052
≥1	40	(82)	39	(80)	
Smoking					
No	19	(39)	20	(41)	0.042
Yes	30	(61)	29	(59)	
Histology					
Non‐adenocarcinoma	24	(49)	21	(43)	0.123
Adenocarcinoma	25	(51)	28	(57)	
T stage					
T1	18	(37)	15	(31)	0.130
T2	31	(63)	34	(69)	
Period					
2011–2017	33	(67)	32	(65)	0.043
2018–2021	16	(33)	17	(35)	
Use of PET					
No	28	(57)	27	(55)	0.041
Yes	21	(43)	22	(45)	
Use of systemic therapy					
No	36	(73)	38	(78)	0.095
Yes	13	(27)	11	(22)	
Performance status					
0–1	42	(86)	43	(88)	0.060
2	7	(14)	6	(12)	
Tumor size (mm)	3.07 (1.01)		3.11 (0.98)		0.041

Abbreviations: CFRT, conventional fractionated radiotherapy; PET, positron emission tomography; PSW, propensity score weighting; SABR, stereotactic ablative radiotherapy; SD, standard deviation.

^a^ Rounded.

**FIGURE 6:**
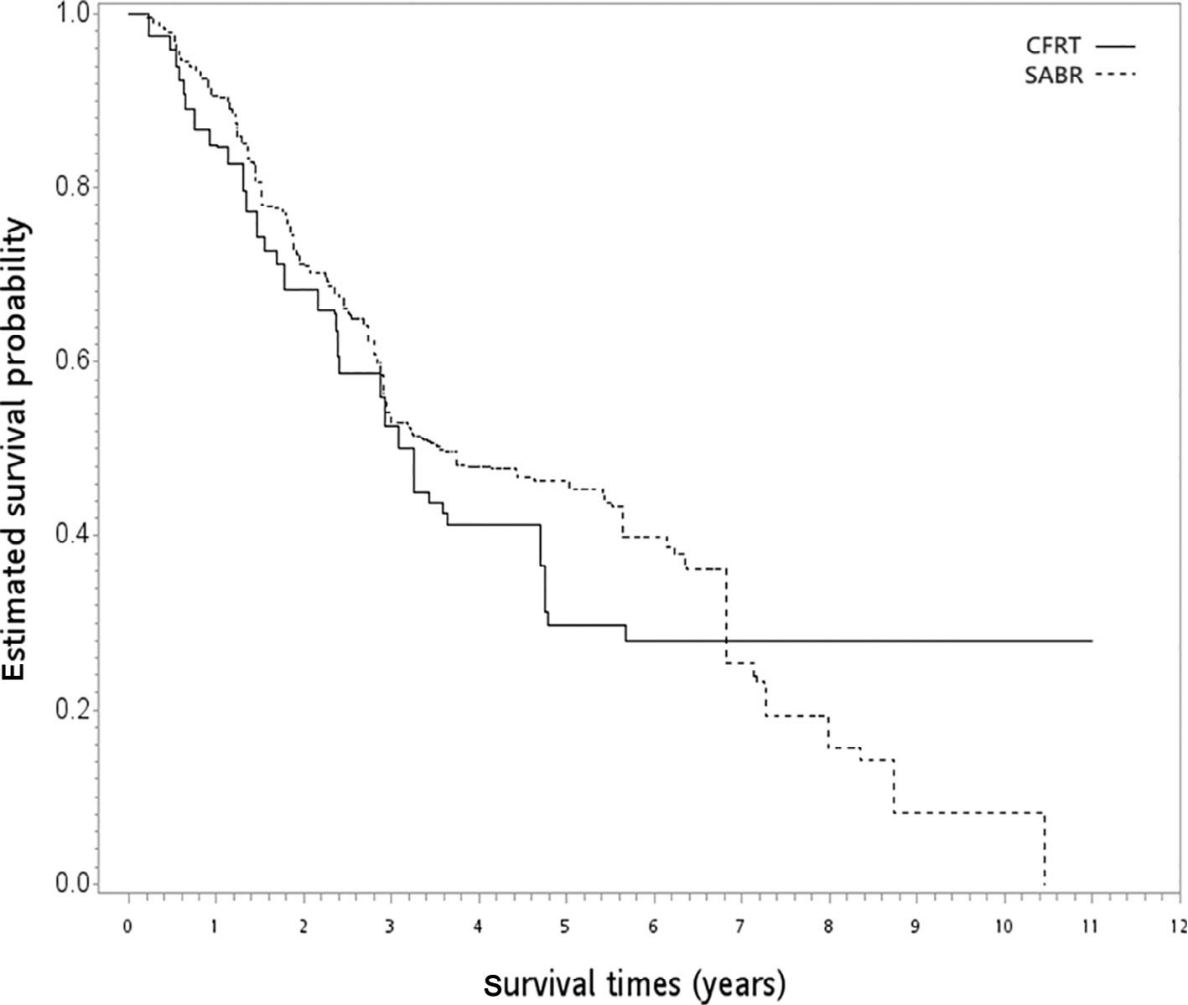
PSW‐adjusted Kaplan–Meier survival curve for SABR versus CFRT in the third supplementary analysis. CFRT, conventional fractionated radiotherapy; PSW, propensity score weighting; SABR, stereotactic ablative radiotherapy.

Among the 303 patients in SA‐3 (Table 5), the PSW‐adjusted HR of death was 0.94 (95% CI 0.63–1.38, *p* = 0.736) when the SABR group was compared to the CFRT group, as seen in Figure 6. ILCM results remained similar (HR = 0.76, *p* = 0.423). The rate of radiation pulmonary toxicity was similarly low (<5%) in both groups and the details were not reported.

**TABLE 5:** Patient characteristics for the study population in the third supplementary analysis.

	SABR (*n* = 252)		CFRT (*n* = 51)		Standardized difference^a^	
	Number or mean (SD)^a^	(%)^a^	Number or mean (SD)^a^	(%)^a^	Before PSW	After PSW
Age (years)	77.89 (8.70)		77.24 (8.15)		0.078	≈0
Sex						
Female	103	(41)	13	(25)	0.331	≈0
Male	149	(59)	38	(75)		
Residency region						
Non‐north	123	(49)	33	(65)	0.325	≈0
North	129	(51)	18	(35)		
Social‐economic status						
Minimum wage or lower	106	(42)	15	(29)	0.266	≈0
Higher	146	(58)	36	(71)		
Comorbidity						
<1	37	(15)	10	(20)	0.131	≈0
≥1	215	(85)	41	(80)		
Smoking						
No	117	(46)	21	(41)	0.106	≈0
Yes	135	(54)	30	(59)		
Histology						
Non‐adenocarcinoma	85	(34)	21	(41)	0.154	≈0
Adenocarcinoma	167	(66)	30	(59)		
T stage						
T1	137	(54)	15	(29)	0.523	≈0
T2	115	(46)	36	(71)		
Period						
2011–2017	106	(42)	34	(67)	0.510	≈0
2018–2021	146	(58)	17	(33)		
Use of PET						
No	74	(29)	28	(55)	0.535	≈0
Yes	178	(71)	23	(45)		
Use of systemic therapy						
No	237	(94)	36	(71)	0.646	≈0
Yes	15	(6)	15	(29)		
Performance status						
0–1	190	(75)	45	(88)	0.338	≈0
2	62	(25)	6	(12)		
Tumor size (mm)	2.87 (0.97)		3.14 (0.98)		0.277	≈0

Abbreviations: CFRT, conventional fractionated radiotherapy; PET, positron emission tomography; PSW, propensity score weighting; SABR, stereotactic ablative radiotherapy; SD, standard deviation.

^a^ Rounded.

Among the 77 patients in SA‐4 (Table 6), PSW‐adjusted HR of death was 0.23 (95% CI 0.01‐4.09, *p* = 0.317) when the SABR group was compared to the CFRT group. The results for ILCM were similar (HR = 0.23, *p* = 0.148). The rate of radiation pulmonary toxicity was similarly low (<5%) in both groups and the details were not reported.

**TABLE 6:** Patient characteristics for the study population in the fourth supplementary analysis.

	SABR (*n* = 68)		CFRT (*n* = 9)		Standardized difference^a^	
	Number or mean (SD)^a^	(%)^a^	Number or mean (SD)^a^	(%)^a^	Before PSW	After PSW
Age (years)	78.74 (7.60)		76.56 (7.38)		0.291	≈0
Sex						
Female	29	(43)	5	(56)	0.260	≈0
Male	39	(57)	4	(44)		
Residency region						
Non‐north	^b^	^b^	^b^	^b^	0.478	≈0
North	^b^	^b^	^b^	^b^		
Social‐economic status						
Minimum wage or lower	^b^	^b^	^b^	^b^	0.447	≈0
Higher	^b^	^b^	^b^	^b^		
Comorbidity						
<1	^b^	^b^	^b^	^b^	0.130	≈0
≥1	^b^	^b^	^b^	^b^		
Smoking						
No	30	(44)	5	(56)	0.230	≈0
Yes	38	(56)	4	(44)		
Histology						
Non‐adenocarcinoma	24	(35)	4	(44)	0.188	≈0
Adenocarcinoma	44	(65)	5	(56)		
T stage						
T1	^b^	^b^	^b^	^b^	0.839	≈0
T2	^b^	^b^	^b^	^b^		
Period						
2011–2017	19	(28)	3	(33)	0.117	≈0
2018–2020	49	(72)	6	(67)		
Use of PET						
No	^b^	^b^	^b^	^b^	0.065	≈0
Yes	^b^	^b^	^b^	^b^		
Use of systemic therapy						
No	^b^	^b^	^b^	^b^	0.188	≈0
Yes	^b^	^b^	^b^	^b^		
Performance status						
0–1	^b^	^b^	^b^	^b^	0.031	≈0
2	^b^	^b^	^b^	^b^		
Tumor size (mm)	2.78 (0.87)		2.79 (1.06)		0.013	≈0

Abbreviations: CFRT, conventional fractionated radiotherapy; PET, positron emission tomography; PSW, propensity score weighting; SABR, stereotactic ablative radiotherapy; SD, standard deviation.

^a^ Rounded.

^b^ The exact numbers are not reported because of a Health and Welfare Data Science Center (HWDC) database center policy to avoid numbers ≤2 in single cells.

In SA‐5, the occurrence of UTI after thoracic radiotherapy was similar in both groups (29% vs. 21% for SABR vs. CFRT, respectively), without any significant difference (PSW‐adjusted odds ratio 1.58, *p* = 0.342), therefore no obvious unmeasured confounding biases were detected during the current analyses.^39^

## DISCUSSION

In this updated retrospective cohort study using the target trial emulation framework, we found that the OS of patients with unresected clinical early‐stage NSCLC treated with SABR did not significantly differ from that of patients treated with CFRT after covariate adjustment in both the primary and supplementary analyses. However, a trend in favor of SABR was observed, which reached significance in the analyses without covariate adjustment. We searched PubMed using the phrase “target trial emulation NSCLC radiotherapy” on March 17, 2024 and found no relevant studies. Therefore, to the best of our knowledge, our study is the first to research this topic using a target trial emulation framework.

Our results were consistent with those of a systematic review published in 2023,^7^ in that there was no significant difference between the efficacies of SABR and CFRT. No additional RCTs have been reported according to a review published in 2024^6^; however, the preliminary results of a relevant ongoing RCT (LUSTRE) reported no significant difference in OS (HR of death 1.18, *p* = 0.4)^37^ between SABR and hypofractionation at 60 Gy/15 Fx. In another RCT, this radiotherapy dose (60 Gy/15 Fx) has been reported to have a similar OS to that of CFRT with 60 Gy/30 Fx for locally advanced NSCLC.^40^ When compared with our earlier research,^10^ this updated study had a similar conclusion, but with a larger sample size (*n* = 351) and an extended follow‐up period >10 years. The crude 5‐year OS (45%) for the SABR arm in our primary analysis may also be compatible with reports by two large institutions in Taiwan.^41^, ^42^ Regarding other studies from Asia included in the above mentioned systematic review,^11^ Karasawa et al. reported adjusted HR of 0.9143 (*p* = 0.69) for OS when accelerated hypofractionated radiotherapy was compared to SBRT,^43^ whereas Tong et al did not report OS.^44^ However, given the non‐randomized nature of this study, our results should be interpreted with caution and results from further studies, especially ongoing RCTs such as LUSTRE, are eagerly awaited. Until more convincing evidence becomes available, our findings can inform shared decision‐making regarding radiotherapy regimens for early stage NSCLC not suitable for surgery, especially for ultra‐central cases where SABR may carry a higher risk of complications.^45^ Considering this, CFRT or hypofractionation could be considered reasonable alternatives, as suggested in the 2024 European guidelines.^46^ Furthermore, for radiotherapy centers not able to perform SABR (such as 42% reported in United Kingdom 2019 survey^47^), CFRT could also be a reasonable alternative.

Our study has several limitations. First, there are always possible unmeasured confounders in retrospective cohort studies like ours, such as tumor location (central vs. peripheral).^45^ There were also possible data accuracy issues in studies using secondary data. Although “TCR is one of the highest‐quality cancer registries in the world,”^14^ it was still not perfect. Furthermore, the choice of treatment was based on actual clinical practice rather than randomization. All the above issues may lead to bias, therefore we utilized the E‐value^32^ to assess the robustness of our findings. Moreover, we used the negative control outcome method^39^ and did not find any obvious unmeasured confounding bias in the current analyses. Second, the sample size of the current study was moderate, which may have led to a wider CI. Third, we did not investigate other potentially relevant endpoints, such as quality of life^48^ and patient‐reported outcomes, owing to limitations in data availability.

## CONCLUSION

In this updated retrospective cohort study using a target trial emulation framework, we found that the OS of patients with early‐stage NSCLC treated with SABR was not significantly different from that of patients treated with CFRT. The results of the relevant ongoing clinical trials are eagerly awaited.

## AUTHOR CONTRIBUTIONS

C.R.C. participated in the concept and design, analysis and interpretation of data, and drafting of the manuscript. H.J.C., W.C.C., C.Y.T., T.C.H., Y.S.L., and H.Y.F. participated in the concept and design, interpretation of data, and drafting of the manuscript. C.C.L. participated in the concept and design, analysis of data and drafting of the manuscript. All authors have read and approved the final version of the manuscript.

## CONFLICT OF INTEREST STATEMENT

All authors declare no conflict of interest.
